# Long-Term Clinical Outcomes after Mix and Match Implantation of Two Multifocal Intraocular Lenses with Different Adds

**DOI:** 10.1155/2019/6789263

**Published:** 2019-01-14

**Authors:** Yuanfeng Jiang, Shaochong Bu, Fang Tian, Jingli Liang, Tiecheng Wang, Xiuli Xing, Hong Zhang, Xiaomin Zhang

**Affiliations:** Tianjin Medical University Eye Hospital, Tianjin Medical University Eye Institute & Tianjin Medical University School of Optometry and Ophthalmology, No. 251, Fukang Road, Nankai District, Tianjin 300384, China

## Abstract

**Purpose:**

To compare long-term clinical outcomes between patients with bilateral implantation of +3.0 diopter (D) multifocal intraocular lenses (IOLs) and mix and match implantation of +2.5 D and +3.0 D multifocal IOLs.

**Material and Methods:**

This retrospective observer-masked cohort study comprised 66 eyes of 33 patients with two different strategies of binocular multifocal IOLs implantation: bilateral +3.0 D (17 patients) (bilateral group) and mix and match +2.5 D and +3.0 D (16 patients) (blended group). Patients were recruited 1 year (±3 months) after second-eye surgery. The primary effectiveness endpoint was binocular uncorrected intermediate visual acuity (UCIVA) at 70 cm. The secondary assessments included binocular visual quality tests and quality-of-vision questionnaire.

**Results:**

The blended group showed clinically better UCIVA (0.10 ± 0.07 logMAR) at 70 cm than the bilateral group (0.26 ± 0.09 logMAR) with a difference of 0.16 ± 0.08 logMAR (*P* < 0.001). Similar binocular visual acuities were achieved between the two groups at the near and far distance. The binocular defocus curves showed better performance in the blended group from 50 cm to 1 m. The mean binocular contrast sensitivities under the photopic conditions with or without glare and mesopic condition without glare were clinically better in the blended group. Both the groups reported low rate of visual phenomena, high rate of spectacle independence, and satisfaction.

**Conclusions:**

Comparing with bilateral implantation of +3.0 D multifocal IOLs during the cataract surgery, mix and match implantation of +2.5 D and +3.0 D multifocal IOLs provides a wider depth of binocular focus, especially for intermediate distances, and better binocular visual quality.

## 1. Introduction

In China, the modern society has been impacted both by an increasingly aged population and rapid improvements of living standard. Traditional cataract surgery had always utilised monofocal intraocular lenses (IOLs), and recent patient demand, however, for spectacle independence postcataract surgery has been fuelled by both a general increase in education and also knowledge gained via a variety of routes including access to information from the World Wide Web [1–3]. To date, there is still no perfect solution to restore the accommodation of the human eye after cataract surgery; however, multifocal IOLs have been commonly used to enable unaided vision over an extended range from distance, through intermediate to near objects. These IOLs are designed to provide multiple focal points simultaneously for distant and near objects, intending to extend the range of functional vision [2, 4, 5].

Current available multifocal IOLs, however, have certain drawbacks. The apodized diffractive designed multifocal IOL, Acrysof IQ ReSTOR +3.0 D, has been proven to achieve satisfactory visual results for both the far and reading distance since its introduction [6–8]. But the +3.0 D lens is not designed to meet the need of intermediate distance, such as computer usage. Additionally, the +3.0 D add produces a perceivable loss of contrast sensitivity and various photic phenomena [8–10]. An alternative model of this lens series, Acrysof IQ ReSTOR +2.5 D, has a lower power addition with less diffractive zones and has been previously reported to provide better intermediate vision along with less glare and halo but with a reduction in efficacy of providing near vision [9, 11–13].

Recently, trifocal IOLs with both good distance and intermediate and near postoperative visual acuity have been used clinically [14–16]. These new IOLs are prohibitively expensive in many countries. For this reason, the bifocal IOLs remain the predominant choices for most patients who wish to be spectacle independent after the surgery. Alternatively, the combination of two different bifocal IOLs with appropriate complementary design patterns has been previously shown to provide excellent depth of binocular focus, while reducing the level of undesirable optical disturbances [17–19].

The current study aimed at providing long-term clinical observation and comparison of the binocular subjective visual performance, spectacle independence, and satisfaction in the two groups of patients: one group was bilaterally implanted with the +3.0 D multifocal IOL and the another was implanted with the +2.5 D multifocal IOL in one eye and the +3.0 D multifocal IOL in the fellow eye.

## 2. Materials and Methods

This was a retrospective cohort study conducted as a postintervention diagnostic evaluation. Voluntary informed consent was obtained from every patient prior to the enrollment. All data for this study were collected and analyzed in accordance with the policies and procedures of the Institutional Review Board of the Tianjin Medical University Eye Hospital and the ethical principles of the Declaration of Helsinki.

### 2.1. Participants

Two groups of patients were studied: the bilateral group consisted of those with bilateral Acrysof IQ ReSTOR +3.0 D IOL implanted in both eyes and the blended group consisted of those with Acrysof IQ ReSTOR +2.5 D IOL implanted in one eye and Acrysof IQ ReSTOR +3.0 D IOL in the fellow eye. Since the poor vision affected by the cataract of the two eyes was not suitable for the test of ocular dominance by the time of surgery, the eye with lower vision or more severe cataract was chosen to be operated on first. The IOL selection strategy was based upon an interview of patients' lifestyle habits, job, and daily activities. All cataract surgeries were performed by the same experienced surgeon (H. Z.) using a standardized procedure consisting of phacoemulsification and primary IOL implantation with 2.2 mm clear corneal incision.

The patients who had uncomplicated cataract surgeries, between January 2016 and July 2017, with appropriate lenses implanted were called and invited to return for one diagnostic test visit 1 year ± 3 months (9 to 15 months) after the operation of the second eye. Exclusion criteria included corneal astigmatism of 1.00 D or higher, irregular corneal astigmatism, myopia of 6 D or higher, amblyopia, previous ocular surgery, and a history of ocular pathologies (e.g., glaucoma and retinopathy). Patients who experienced any intraoperative or postoperative complications that could compromise the visual function were not considered eligible for this study.

### 2.2. Clinical Outcomes and Assessments

The difference in binocular uncorrected intermediate visual acuity (UCIVA) of the bilateral group versus the blended group was considered as the primary effectiveness endpoint. Secondary endpoints assessed included: (1) binocular uncorrected/corrected distance visual acuity (UCDVA/CDVA), distance corrected intermediate visual acuity (DCIVA), uncorrected/distance corrected near visual acuity (UCNVA/DCNVA), (2) binocular defocus curve, (3) binocular contrast sensitivity, and (4) quality-of-vision questionnaire.

Visual acuity testing was performed with 100% contrast E Standard Logarithmic Visual Acuity chart at far (5 m), near (UCNVA at preferred distance and DCNVA at 40 cm), and intermediate (70 cm) distances under photopic lighting conditions (>85 cd/m^2^). All the evaluations of visual acuity with a difference greater than or equal to 0.1 logMAR, denoting a clinical equivalent of 1 line on the Early Treatment Diabetic Retinopathy Study chart, were considered to be clinically significant.

The defocus curve was obtained by measuring the binocular visual acuity when reading the logMAR chart at 5 m under full refractive correction. To produce defocusing, a sequential progression of lenses with an increment of −0.50 D was used one at a time. The range of this sequence was from +1.5 to −5.0 D. At each step, logMAR acuity was measured and recorded.

Binocular distance contrast sensitivity at 3, 6, 12, and 18 cycles per degree (CPD) was measured using the CSV-1000E chart (Vector Vision, Greenville, OH) at a distance of 8 feet using the patient's spectacle corrections. Patients were tested with and without glare (135 lux for photopic glare and 28 lux for mesopic glare) under photopic (85 cd/m^2^) and mesopic (3 cd/m^2^) conditions. Patients were dark, adapted for 10 minutes before mesopic testing. Raw scores were converted to log units. Mean differences over 0.3 log units were considered to be clinically significant when they occurred at 2 or more spatial frequencies based on ANSI Z80.12-2007 and IS EN ISO 11979-9:2006 [20, 21].

During the visit, all patients completed a concise postoperative quality-of-vision (QoV) questionnaire consisting of optical disturbances (halo/glare), spectacle dependence, and satisfaction; each was assessed using 3 levels. For halo/glare, the levels were none, slight, and severe; for spectacle dependence, the levels were none, sometimes, and always; and for satisfaction, the levels were satisfied, fair, and unsatisfied.

### 2.3. Statistical Analysis

Sample size estimates for this study were based on the primary outcome measure of UCIVA using Power Analysis and Sample Size (PASS 15) statistical software (Kaysville, Utah, USA). For *α* = 0.05 and 1 − *β* = 0.90, a sample size of 15 patients per group was sufficient to detect a mean difference of 0.1 logMAR in UCIVA of bilateral group versus blended group with the two-sample *t*-test. Anticipating a 13% loss in case some of the patients failed to finish all the tests of the study, 17 patients per group should be enrolled.

All statistical analyses were analyzed using Number Cruncher Statistical System (NCSS 11) statistical software (Kaysville, Utah, USA). Two-sided *P* values less than 0.05 were considered statistically significant.

Differences between the groups in continuous variables were compared using the two-sample *t*-test (equal-variance *t*-test or Aspin-Welch unequal-variance *t*-test). Categorical variables were compared using the Fisher's exact test or the Mann–Whitney *U* test, where appropriate.

## 3. Results

### 3.1. Patient Disposition and Demographics

Of the 34 patients in the prepared list who were qualified and consented to participate in this retrospective cohort study, 33 patients who completed all the tests were enrolled with 17 in the bilateral group and 16 in the blended group. Age, gender, preoperative spherical equivalent, CDVA, axial length, IOL diopter, target refraction, and the time after surgery did not differ significantly between groups (Table 1).

### 3.2. Bilateral Visual Acuity

Table 2 shows the postoperative binocular visual acuity outcomes. Primary study efficacy showed that the mean (standard deviation (SD)) binocular UCIVA at 70 cm was 0.26 ± 0.09 logMAR in the bilateral group and 0.10 ± 0.07 logMAR in the blended group, with a statistically and clinically significant difference of 0.16 ± 0.08 logMAR (95% CI, 0.10–0.22). After being corrected for distance, the binocular intermediate visual acuity was still clinically better in the blended group compared to the bilateral group (mean difference, 0.15 ± 0.09 logMAR). The mean preferred reading distance of the bilateral group was a little closer than the blended group with a statistically significant difference (mean difference, −3.35 ± 4.08 cm). Similar results were found in the mean binocular UCDVA, CDVA at 5 m, DCNVA at 40 cm, UCNVA at preferred reading distance, and the postoperative spherical equivalent between the two groups.

### 3.3. Defocus Curve

**Figure 1:**
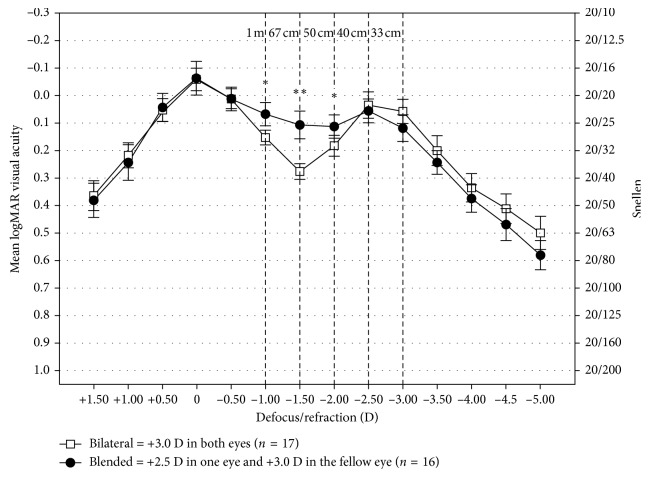
Mean and 95% confidence limits for binocular vision for defocus curves testing (D, diopter; ^*∗*^ *P* < 0.05; ^*∗∗*^ *P* < 0.05 and mean difference > 0.1 logMAR).

The binocular defocus curves showed that both the groups provided full range of functional vision from near to distance; the patients achieved 0.3 logMAR or better binocular vision from +1.00 to −3.50 D of defocus (Figure 1). The defocus curve of the bilateral group showed the expected logMAR bimodal peak pattern, with a distance visual acuity peak (−0.06 ± 0.08 logMAR) at 0.00 D and a near visual acuity peak (0.04 ± 0.09 logMAR) at −2.50 D (approximately 33 cm) of defocus. The blended group showed better binocular visual acuity at −1.00, −1.50, and −2.00 D (approximately, 1 m, 67 cm, and 50 cm) compared to the bilateral group, and the differences were statistically significant (*T* = 3.640, 6.414, and 2.616; *P*=0.001, <0.001, and  0.014). No significant differences were found between the two groups at other defocus points.

### 3.4. Binocular Contrast Sensitivity

**Figure 2:**
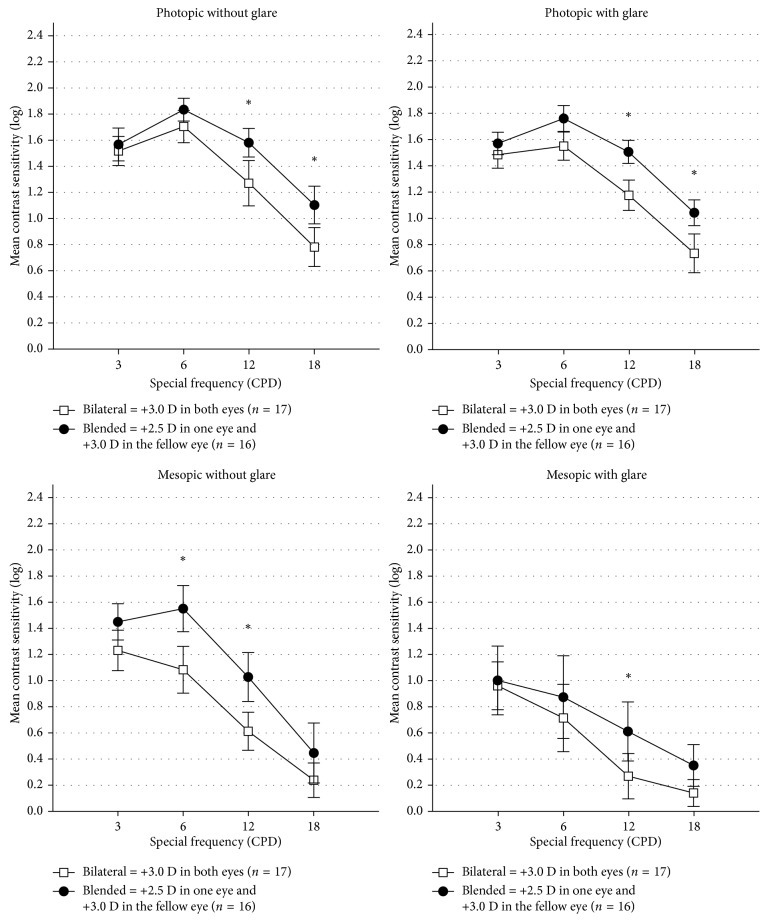
Mean and 95% confidence limits for binocular contrast sensitivity testing under photopic and mesopic conditions with or without glare. CPD, cycles per degree; ^*∗*^ *P* < 0.05 and mean difference > 0.3 log unit.

Clinically relevant differences (mean difference > 0.3 log unit occurred at 2 or more spatial frequencies) of binocular contrast sensitivity were shown under photopic conditions with (0.31 ± 0.28 at 12 CPD and 0.32 ± 0.28 at 18 CPD) or without (0.33 ± 0.20 at 12 CPD and 0.31 ± 0.24 at 18 CPD) glare and mesopic conditions without glare (0.47 ± 0.34 at 6 CPD and 0.42 ± 0.32 at 12 CPD), between blended group and bilateral group (Figure 2). A difference of 0.34 ± 0.38 log unit was shown at 12 CPD under mesopic condition with glare between groups.

### 3.5. Questionnaire

**Figure 3:**
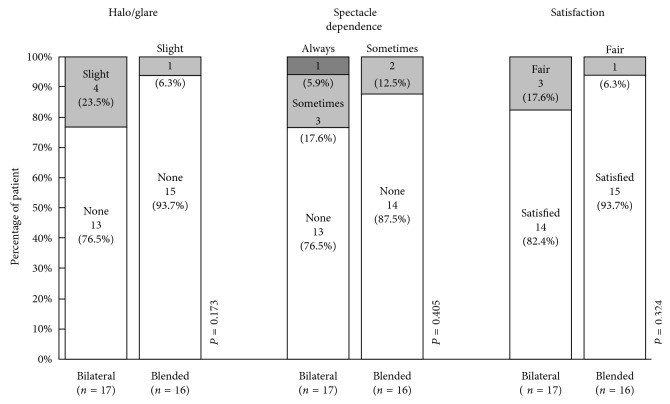
Concise postoperative quality-of-vision questionnaire. Levels: halo/glare, none/slight/severe; spectacle dependence, none/sometimes/always; and satisfaction, satisfied/fair/unsatisfied.

Four patients reported slight halo or glare in the bilateral group while only one patient in the blended group (Figure 3). None of the patients reported severe symptom in either group. There were two patients who required the assistance of reading spectacles occasionally in the blended group, while glasses were needed for three patients sometimes and one patient all the time in the bilateral group, especially during computer usage. 82.4% of patients in the bilateral group and 93.7% of the blended group were completely satisfied with their postoperative visual quality. However, there were no statistically significant differences in the three questionnaires.

## 4. Discussion

Ever since the introduction of multifocal IOLs, variety of models and strategies were devoted to extend the range of vision after cataract surgery and to reduce the dependence on glasses. Among which, the AcrySof IQ ReSTOR family of IOLs provides two models of multifocal IOLs with different near adds [6, 12, 22]. Studies have shown that, in addition to the excellent binocular distance visual acuity produced by both IOLs, ReSTOR +3.0 D has a greater advantage in near vision while ReSTOR +2.5 D yields better results in the range of intermediate distance [6, 11, 12].

Traditionally, except for far vision, multifocal IOLs with proper near additions can provide adequate near vision for reading and writing with fairly high spectacle independence and postoperative satisfaction [4, 6, 8]. However, with the frequent usage of smartphones, computers, and tablets being involved in the daily life of the modern elderly, demands for intermediate distance vision have become increasingly prominent. The attempt of combining different models of multifocal IOLs asymmetrically, so-called the mix and match method, was introduced in the 1990s, intending to improve the range and quality of binocular vision by complementing each other's advantages [23, 24].

In this cohort study, mix and match implantation of two different additional power multifocal IOLs resulted in significantly better UCIVA without sacrificing the excellent UCNVA. All of the patients achieved a wide range of vision one year after the surgery, whether bilateral implanted with the Restor +3.0 D multifocal IOL in both the eyes or the Restor +2.5 D and +3.0 D multifocal IOL contralaterally in each eye. Recently, Nuijts et al. conducted a parallel group study to access the visual outcomes of blended implantation of the ReSTOR +2.5 D and +3.0 D multifocal IOL and bilateral implantation of ReSTOR +2.5 D multifocal IOLs [17]. They found similar results in distance and intermediate vision between the groups, while the blended group showed better performance in near vision. In order to exclude the possible influence caused by biometric error and difference of postoperative refractive status, we compared the predictive target refraction and the postoperative spherical equivalent between the two groups and obtained negative results. In addition, after being corrected for distance, DCIVA remained better in the blended group, while there was still no difference in DCNVA between the two groups.

In particular, the binocular defocus curve of the blended group propped up a wider and higher platform between the two logMAR peaks at near and distance presented in both the groups, which were also consistent with the previous studies [10, 12, 17]. Visual acuity was almost 2 lines better (mean difference ± SD and 0.17 ± 0.08 logMAR) in the blended group than in the bilateral group at −1.50 D (67 cm) and approximately 1 line better (0.08 ± 0.07 and 0.07 ± 0.08 logMAR) at −1.00 D (1 m) and −2.00 D (50 cm) of defocus. The binocular defocus curve of mix and match implantation of bifocal IOLs demonstrated a similar trifocal behavior compared with binocular implantation of the trifocal IOLs [18, 19]. Such a combination remedied the limitation of visual function from 50 cm to 1 m after bifocal IOLs implantation. And this might result in fewer patients relied on glasses in the blended group, especially in the intermediate distances scenarios.

The current study suggests that the blended implantation of multifocal IOL could improve the postoperative contrast sensitivity of the patients compared to the bilateral +3 D group. Our results showed that the patients in the blended group achieved better results under the photopic conditions at medium and high spatial frequencies with or without glare, as well as the mesopic condition at medium spatial frequencies without glare. These favourable outcomes might be attributed to the different ideal of design and complementary strengths of these two IOLs. Nuijts et al. reported similar binocular results at all spatial frequencies under photopic conditions with and without glare between the blended group and the bilateral +2.5 D implanted group [17]. Furthermore, Vilar et al. found that the blended group performed better at 3, 6, and 12 CPD under mesopic conditions with glare than the bilateral trifocal group (Acrysof PanOptix TFNT00) [19]. However, a small RCT showed that there was no significant difference in binocular contrast sensitivity between mix and match implantation of these two bifocal IOLs and bilateral implantation of a trifocal IOL (FineVision) [18]. These data, from our and other studies, suggested an equal or superior clinical effect of such a combination in comparison with other strategies with respect to the binocular contrast sensitivity.

Although mostly in the normal range, the loss of contrast sensitivity and optical disturbance still remain the main common recognized drawbacks of multifocal IOLs [2, 5]. The loss of contrast sensitivity can partially be explained by the division of available light energy into the eye between 2 or more focal points [6, 25]. Due to the difference in design, ReSTOR +3.0 D combines a 0.86 mm diameter central diffractive zone and 9 diffractive steps in 10.2 mm^2^ area for enhanced near vision close to 40 cm, surrounded by a refractive region for distance vision, while ReSTOR +2.5 D has 7 diffractive steps in smaller area (8.4 mm^2^) with a 0.94 mm diameter central refractive zone dedicated 100% to distance. Under normal circumstances, binocular measurements are better than monocular [26]. Thus, the mutual compensation of asymmetric light distribution through the mix and match approach might allow the binocular contrast sensitivity to be superior to the bilateral group with symmetrical light distribution.

Another potential risk of multifocal IOLs affecting the visual quality and the quality of life is the unwanted optical phenomena. Similar to the previous studies, the simple questionnaire in this study tentatively indicated a low rate of halo or glare and high level of patient satisfaction in both the groups, while the mix and match approach seemed to be more efficient in avoiding visual disturbances [17]. One of the hypotheses was the different design of the +2.5 D model with fewer rings and wider diffractive steps, which had been found to exhibit better objective monocular visual quality with lower amount of higher-order aberrations and less intraocular scattering than the +3.0 D model in other and our studies (supplementary materials), might contributed to the better binocular performance when being combined with the +3.0 D [12, 17, 27]. Unfortunately, there is still no instrument or method to detect the objective binocular visual quality directly. And further studies with a larger number of patients are required to verify the reliability of this theory.

A limitation of this study was the lack of a control group with a trifocal IOL bilaterally implanted in order to assess the differences in terms of intermediate visual quality and contrast sensitivity comparing with the blended group. Although several models of trifocal IOLs have been widely used in the western countries, there is only one of them (AT Lisa tri839MP) commercially and clinically available in China, which is more than three times the price of the bifocal IOLs. Therefore, it is equally important to find a strategy that can provide both excellent full range of visual function and high cost performance. Furthermore, the results of this study were possibly biased by the nonrandomization and the limited number of patients enrolled, which would reduce the statistical power of the analysis. As the selection of the implantation strategy was based on the individual's lifestyle, patient's subjective intention and choice of the strategy might introduce some bias into the subjective clinical outcomes, especially the satisfaction and spectacle dependence questionnaire. But because this approach of IOL selection is in accordance with clinical practice and patients' interests, we consider it a positive feature of the study design. However, a randomized prospective trial with larger sample is required to confirm the results of this study.

## 5. Conclusions

In conclusion, the mix and match implantation of AcrySof IQ ReSTOR +2.5 D and +3.0 D multifocal IOLs resulted in better intermediate visual acuity compared with bilateral implantation of ReSTOR +3.0 D multifocal IOLs during cataract surgeries, without sacrificing the visual acuity for near and far at one year after the operation. In addition, the mix and match approach of these two lenses with different near adds showed better binocular contrast sensitivities. Subjectively, halo and glare were well tolerated while high rates of spectacle independence, and satisfaction were reported in both the combination modes. Therefore, our results suggested an alternative strategy of multifocal IOLs selection for patients who require high quality of vision from far to near without spectacles.

## Figures and Tables

**Table 1:** Patient characteristics of the two groups.

Parameter	Mean ± SD		
	Total	Bilateral	Blended
No. of patients (eyes)	33 (66)	17 (34)	16 (32)
Age (years)^a^	67.09 ± 8.46	66.59 ± 6.23	67.63 ± 10.52
Gender (female/male)	18/15	9/8	9/7
Spherical equivalent (D)	−1.63 ± 2.23	−1.87 ± 2.27	−1.38 ± 2.39
Preoperative CDVA	0.49 ± 0.38	0.53 ± 0.46	0.47 ± 0.26
Time since surgery (months)	11.79 ± 2.12	11.71 ± 2.20	11.88 ± 2.09
Axial length (mm)	23.84 ± 1.50	23.98 ± 1.63	23.72 ± 1.52
IOL implanted (D)	19.95 ± 3.80	19.56 ± 4.74	20.46 ± 2.67
Target refraction (D)	−0.15 ± 0.19	−0.17 ± 0.22	−0.14 ± 0.18

SD,  standard deviation; D, diopter. ^a^The difference of gender was evaluated using Fisher's exact test. All other comparisons were performed with two-sample *t*-test at *P* < 0.05. Statistically significant differences were not found for any of the tested parameters.

**Table 2:** Postoperative data of the two groups.

Parameter^a^	Mean ± SD (95% CI)			*T*-statistic	Prob level
	Bilateral	Blended	Difference		
Binocular visual acuity (logMAR)^b^					
UCDVA	−0.02 ± 0.10 (−0.07, 0.03)	−0.00 ± 0.12 (−0.06, 0.06)	−0.02 ± 0.11 (−0.10, 0.05)	−0.618	0.541
CDVA	−0.08 ± 0.07 (−0.12, −0.04)	−0.07 ± 0.11 (−0.13, −0.01)	−0.01 ± 0.09 (−0.08, 0.05)	−0.427	0.672
UCIVA	0.26 ± 0.09 (0.21, 0.30)	0.10 ± 0.07 (0.06, 0.14)	0.16 ± 0.08 (0.10, 0.22)	5.660	<0.001
DCIVA	0.26 ± 0.08 (0.22, 0.30)	0.11 ± 0.09 (0.06, 0.16)	0.15 ± 0.09 (0.09, 0.21)	5.079	<0.001
DCNVA	0.04 ± 0.08 (−0.01, 0.08)	0.06 ± 0.10 (0.00, 0.11)	−0.02 ± 0.09 (−0.08, −0.04)	−0.686	0.498
UCNVA	0.04 ± 0.08 (−0.01, 0.08)	0.07 ± 0.10 (0.01, 0.12)	−0.07 ± 0.09 (−0.14, −0.00)	−1.063	0.296
Preferred reading distance (cm)	33.59 ± 3.91 (31.58, 35.60)	36.94 ± 4.27 (34.66, 39.21)	−3.35 ± 4.08 (−6.25, −0.45)	−2.354	0.025
Spherical equivalent (D)	−0.24 ± 0.35 (−0.36, −0.12)	−0.16 ± 0.31 (−0.27, −0.05)	−0.08 ± 0.33 (−0.24, 0.08)	−1.019	0.312

SD, standard deviation; CI, confidence Interval; D, diopter. ^a^All parameters were evaluated by the two-sample *t*-test and were considered to be statistically significant at *P* < 0.05. ^b^The visual acuity with a mean difference ≥0.1 logMAR and *P* < 0.05 was considered to be clinically significant.

## Data Availability

The data used to support the findings of this study are available from the online repository of Mendeley Data (DOI: https://data.mendeley.com/datasets/g8vw3tb56k/draft?a=9c3c15ed057d-416b-b82a-9da8bd8829a2) or from the corresponding author upon request.

## Supplementary Materials

Supplementary Materials Objective monocular visual quality of +2.5 D implanted eyes and +3.0 D implantedeyes in the blended group were assessed by modulation transfer function (MTF) cutofffrequency, Strehl ratio (SR), objective scatter index (OSI) and wavefront aberrations.
