# Supplementary material for: Long-Term Clinical Outcomes after Mix and Match Implantation of Two Multifocal Intraocular Lenses with Different Adds
Source: J Ophthalmol. 2019 Jan 14;2019:6789263. doi: 10.1155/2019/6789263 (PMC6348908; doi:10.1155/2019/6789263)
Supplement: Supplementary Materials — Objective monocular visual quality of +2.5 D implanted eyes and +3.0 D implantedeyes in the blended group were assessed by modulation transfer function (MTF) cutofffrequency, Strehl ratio (SR), objective scatter index (OSI) and wavefront aberrations. [file 6789263.f1.pdf]

Supplementary material

Objective monocular visual quality of +2.5 D implanted eyes and +3.0 D implanted eyes in the blended group were assessed by modulation transfer function (MTF) cutoff frequency, Strehl ratio (SR), objective scatter index (OSI) and wavefront aberrations.

Methods

MTF cutoff, SR and OSI were measured by OQASII (Visiometrics SL, Terrassa, Spain), a double-pass system which obtains images from a near infrared light point-source object reflecting on the retina. The MTF cutoff is the cutoff frequency (c/deg) at 1% of maximum MTF which indicating the spatial frequency corresponding to the contrast of the retinal image at 1% of the original scene. The SR provided by OQASII is calculated in two dimensions as the ratio between the area under the MTF curve of the measured eye and that of the ideal eye (aberration-free). A SR (ranges from 0 to 1.0) of 1.0 therefore indicates a perfect optical system. The OSI is the ratio of light intensity in the intensity curve between the annular area within 12 and 20 minutes of arc and that within 1 minute of arc of the central peak. All measurements were conducted and recorded with a 4.0 mm (only if pupil's diameter larger than 4 mm) artificial pupil. The higher the value of the MTF cutoff and SR, and the lower the OSI, the better the optical quality.

Wavefront and corneal topography were captured by the iTrace (Tracey Technologies, Houston, TX, USA) in all eyes under mesopic conditions (3 cd/m^2). The root mean square (RMS) values of spherical aberration, coma, trefoil and total higher-order aberrations (HOA) from the whole ocular at central 4.0 mm diameter (only if pupil's diameter larger than 4 mm) were recorded and compared between +2.5 D implanted eyes and +3.0 D implanted ones. The RMS was expressed in micrometer (μm).

Results

Objective monocular visual quality

The grouping outcomes of the OQAS and iTrace parameters at 4.0 mm pupillary diameter were summarized in Table 1. As shown, there was a statistically significant lower value of OSI in the +2.5 D implanted eyes (1.20±0.37) than in the +3.0 D ones (1.83±0.61); while the mean values of MTF cutoff and SR were higher in the +2.5 D implanted eyes than in the +3.0 D ones without statistically significant differences being concluded. As for postoperative ocular aberrations, although no statistically significant differences were found in spherical aberration, trefoil and total HOA

across the two groups except for coma (P = 0.038), they all tended to zero for 4.0 mm pupil.

Table 1. Objective monocular visual quality.

	Mean ± SD (95% CI)			
Parameter	+2.5 D	+3.0 D	Difference	Prob level
OQAS	(n=15)	(n=15)		
MTF cutoff (c/deg)	34.64±10.71<br>(28.71, 40.57)	28.28±8.16<br>(23.75, 32.80)	6.36±9.52<br>(-0.76, 13.48)	0.078
SR	0.20±0.07<br>(0.17, 0.24)	0.18±0.07<br>(0.14, 0.21)	0.03±0.07<br>(-0.02, 0.08)	0.298
OSI	1.20±0.37<br>(1.00, 1.40)	1.83±0.61<br>(1.49, 2.17)	-0.63±0.50<br>(-1.00,-0.25)	0.002*
HOAs (μm)	(n=12)	(n=12)		
Spherical	0.01±0.02<br>(-0.01, 0.02)	-0.03±0.15<br>(-0.12, 0.07)	0.04±0.15<br>(-0.06, 0.13)	0.398
Coma	0.09±0.07<br>(0.05, 0.13)	0.17±0.11<br>(0.10, 0.24)	-0.08±0.13<br>(-0.16, -0.00)	0.038*
Trefoil	0.16±0.02<br>(0.11, 0.20)	0.23±0.26<br>(0.06, 0.39)	-0.07±0.27<br>(-0.24, 0.10)	0.381
Total	0.23±0.11<br>(0.16, 0.30)	0.36±0.30<br>(0.17, 0.55)	-0.13±0.32<br>(-0.33, 0.07)	0.187

SD = Standard deviation; CI = Confidence Interval; MTF cutoff = modulation transfer function cutoff frequency; c/deg = cycles per degree; SR = Strehl ratio; OSI = objective scatter index; HOAs = higher-order aberrations; All comparisons were performed with Two-Sample T-Test; * $P<0.05$;
